# Carinatines A and B, *Lycopodium* Alkaloids from *Phlegmariurus carinatus*

**DOI:** 10.1007/s13659-014-0030-6

**Published:** 2014-07-17

**Authors:** Fei Liu, Yu-Cheng Liu, Wei-Wei Jiang, Juan He, Xing-De Wu, Li-Yan Peng, Jia Su, Xiao Cheng, Qin-Shi Zhao

**Affiliations:** 1 State Key Laboratory of Phytochemistry and Plant Resources in West China, Kunming Institute of Botany, Chinese Academy of Science, Kunming, 650201 People’s Republic of China; 2 University of the Chinese Academy of Sciences, Beijing, 100049 People’s Republic of China

**Keywords:** *Lycopodium* alkaloid, *Phlegmariurus carinatus*, Carinatines A and B, Acetylcholinesterase (AChE) inhibitory activity

## Abstract

Carinatine A (**1**), a C_16_N_2_-type *Lycopodium* alkaloid possessing a 5/6/6/6 ring system formed by a new C-4/C-12 bond, and carinatine B (**2**), the first derivative of lycojaponicumin C, along 16 known compounds, were isolated from the whole plant of *Phlegmariurus carinatus*. Their structures were elucidated based on the spectroscopic data. The two new isolates were no inhibitory activity for the acetylcholinesterase (AChE).

## Introduction

The *Lycopodium* alkaloids, possessing diverse skeletons and interesting biological activities, have attracted great interest from biogenetic, synthetic and biological perspectives [1–7]. There are over 500 species in Lycopodiales around the world, but just about 50 species phytochemically have been studied. Now, more than 300 alkaloids have been reported [1–4].

**Fig. 1:**
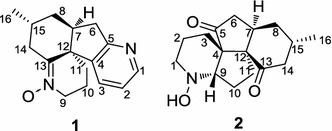
Chemical structures of isolated compounds **1**–**2**

*Phlegmariurus carinatus* has been historically used as a traditional Chinese herbal medicine for the treatment of rheumatism, swelling, and pain [8, 9]. No *Lycopodium* alkaloids have been reported from this plant. During our continuing search for structurally interesting and bioactive *Lycopodium* alkaloids [10–15], two new *Lycopodium* alkaloids, carinatines A (**1**) and B (**2**), along with 16 known compounds, were isolated from the whole herb of *P. carinatus*. Carinatine A (**1**) was a C_16_N_2_-type *Lycopodium* alkaloid possessing a 5/6/6/6 ring system formed by a new C-4/C-12 bond. Carinatine B (**2**), a new C_16_N-type *Lycopodium* alkaloid with a 5/5/6/6 tetracyclic ring system, was the first derivative of lycojaponicumin C [16]. Herein, we report the isolation and structure elucidation of these isolates (Fig. 1).

## Results and Discussion

**Fig. 2:**
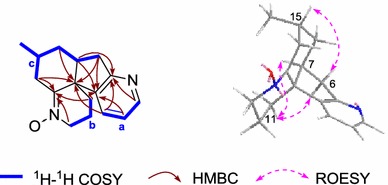
Selected 2D NMR correlations for carinatine A (**1**)

Carinatine A (**1**) was obtained as a colorless oil and its molecular formula was established to be C_16_H_20_N_2_O by HREIMS at *m*/*z* 256.1579 [M]^+^ (calcd 256.1576), indicating 8° of unsaturation. Analysis of the ^1^H and ^13^C NMR spectra of **1** revealed 16 carbon signals due to four quaternary carbons, five tertiary carbons, six methylenes, and one methyl group (Table 1). Among them, two sp^2^ quaternary carbons were attributable to two amide groups (*δ*_C_ 165.0 and 156.2). The ^1^H-^1^H COSY cross-peaks of **1** disclosed the presence of three structural fragments, **a** (C-1–C-3), **b** (C-9–C-11), and **c** (C-6–C-8–C-15–C-14 and C-15–C-16), as shown in Fig. 2. According to the HMBCs from H-1 and H-3 to C-5 (*δ*_C_ 165.0) and from H-2 to C-4 (*δ*_C_ 141.8) indicated the presence of a pyridine ring which was formed by the connectivities of C-1 and C-5 through a nitrogen atom. While the connectivities between C-9 (*δ*_C_ 59.0) and C-13 (*δ*_C_ 156.2) through a nitrogen atom was revealed by the HMBC cross-peak from H-9 to C-13. An HMBCs from H-7 to C-5 indicated the connectivity of C-5 and C-6. Finally, the connectivity of C-13 and C-14 and the connectivities of C-4, C-7, C-11, and C-13 through C-12 were elucidated by HMBCs from H-11 and H-14 to C-13 and from H-3, H-6, H-8, H-10, and H-14 to C-12. Thus, the gross structure of **1** was established (Fig. 1).

**Table 1:** ^1^H (600 MHz) and ^13^C NMR (150 MHz) data of **1** and **2** (*δ* in ppm, *J* in Hz)

No.	1^a^		2^a^	
	*δ*_H_ (mult, *J*, Hz)	*δ*_C_ (mult)	*δ*_H_ (mult, *J*, Hz)	*δ*_C_ (mult)
1a	8.30 (1H, dd, 5.3, 1.3)	149.5 d	3.16 (1H, m)	60.2 t
1b			2.08 (1H, m)	
2a	7.18 (1H, dd, 7.7, 5.1)	123.3 d	2.30 (1H, m)	22.9 t
2b			1.49 (1H, m)	
3a	7.43 (1H, dd, 7.7, 1.3)	134.2 d	1.80 (1H, d, 12.8)	32.8 t
3b			0.97 (1H, m)	
4		141.8 s		62.4 s
5		165.0 s		220.5 s
6a	3.48 (1H, dd, 17.3, 7.1)	40.7 t	2.61 (1H, dd, 18.8, 8.3)	47.0 t
6b	2.60 (1H, d, 17.3)		2.14 (1H, overlapped)	
7	2.66 (1H, q, 7.1)	45.3 d	2.38 (1H, overlapped)	40.0 d
8a	1.50 (1H, dt, 14.0, 7.1)	38.4 t	1.65 (1H, dd, 10.0, 3.3)	37.5 t
8b	1.36 (1H, ddd, 14.0, 7.1, 3.1)		1.65 (1H, dd, 10.0, 3.3)	
9	2.38 (2H, m)	59.0 t	2.24 (1H, m)	76.8 d
10a	1.91 (1H, overlapped)	19.5 t	2.01 (1H, ddd, 12.4, 8.4, 6.2)	29.8 t
10b	1.83 (1H, m)		1.28 (1H, m)	
11a	1.92 (1H, overlapped)	32.8 t	2.74 (1H, ddd, 13.8, 10.8, 9.0)	33.2 t
11b	1.75 (1H, m)		1.42 (1H, dd, 13.8, 9.8)	
12		52.5 s		66.0 s
13		156.2 s		214.0 s
14a	2.94 (1H, dd, 17.1, 7.1)	33.9 t	2.38 (1H, dd, 16.6, 6.1)	49.0 t
14b	2.01 (1H, m)		2.28 (1H, dd, 14.1, 4.9)	
15	1.60 (1H, m)	27.9 d	2.27 (1H, m)	30.2 d
16	0.84 (3H, d, 6.8)	20.1 q	0.93 (3H, d, 7.2)	19.6 q

^a^Recorded in methanol-*d*_4_

The relative configuration of **1** was established by the ROESY spectrum (Fig. 2). The correlations of H-6a and H-7 with H-11 and of H-6b with H-15 indicated H-7 was *α*-oriented, while H-15 was *β*-oriented. Therefore, the structure of **1**, named carinatine A, was elucidated as a new C_16_N_2_-type *Lycopodium* alkaloid with a 5/6/6/6 tetracyclic ring system (Fig. 1).

**Fig. 3:**
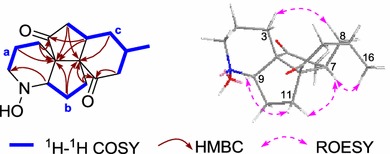
Selected 2D NMR correlations for carinatine B (**2**)

Carinatine B (**2**), a white amorphous powder, had a molecular formula C_16_H_23_NO_3_ as established by HREIMS at *m*/*z* 277.1667 [M]^+^ (calcd 277.1678), suggesting 6° of unsaturation. IR absorptions implied the existence of hydroxy (3425 cm^−1^) group. Analysis of the 1D and 2D NMR spectra revealed the existence of 16 carbons due to two carbonyl carbons (*δ*_C_ 220.5 and 214.0), two *sp*^3^ quaternary carbons (*δ*_C_ 62.4 and 66.0), three *sp*^3^ methines (*δ*_C_ 30.2, 40.0, and 76.8), eight *sp*^3^ methylenes, and one methyl (*δ*_H_ 0.93; *δ*_C_ 19.6; Table 1). The ^1^H-^1^H COSY correlations revealed the existence of three fragments, **a** (C-1–C-3), **b** (C-9–C-11), and **c** (C-6–C-8–C-15–C-14, C-15–C-16), as shown in Fig. 3. In the HMBC spectrum (Fig. 3), the correlations from H-11 and H-14 to C-13 (*δ*_C_ 214.0) and from H-8, H-10, and H-11 to C-12 (*δ*_C_ 66.0) indicated the connection of C-13 with C-14 (*δ*_C_ 49.0), and the connections of C-7 (*δ*_C_ 40.0), C-11 (*δ*_C_ 33.2) and C-13 through C-12. While the connectivities of C-1 (*δ*_C_ 60.2) and C-9 (*δ*_C_ 76.8) through a nitrogen atom was revealed by HMBC from H-9 to C-1. At last, the HMBCs from H-3 and H-7 to C-5 combining with the HMBCs of H-2, H-3, H-6, H-7, and H-10 to C-4 (*δ*_C_ 62.4) constructed the linkage of C-5 (*δ*_C_ 220.5) and C-6 (*δ*_C_ 47.0) and the connections of C-3, C-5, C-9, and C-12 through C-4. Therefore, the planar structure of **2** was established as a new C_16_N-type *Lycopodium* alkaloid with a 5/5/6/6 tetracyclic ring system, the first derivative of lycojaponicumins C [16].

In ROESY spectrum (Fig. 3), the cross peaks of H-7 and H-11b with H-16, H-9 with H-11a and H-3 with H-8 were observed, which suggested that H-9 and H-15 were *β*-oriented and H-7 was *α*-oriented. Therefore, the structure of compound **2** was elucidated as show in Fig. 1 and named as carinatine B.

Carinatines A (**1**) and B (**2**) were tested for AChE inhibitory activities using the Ellman method reported previously [17]. However, none of them showed obvious activity.

The known compounds, compared with literatures data, were identified as 8-deoxy-13-dehydroserratinine [18], lobscurinol [19], lycoflexine [20], *N*-oxide-lycoflexine [21], fawcettimine [22], lycoposerramine-Q [23], phlegmariurine B [23], phlegmariurine A [24], obscurinine [24], lycopodine [25, 26], lycodoline [27, 28], lucidioline [29], lycopodatine C [30], gnidioidine [31], malycorins B [32], and lycodine [33].

## Experimental Section

### Plant Material

The whole plants of *P. carinatus* were collected from Guangxi Province, P. R. China, and identified by Professor Xiao Cheng. A voucher specimen (voucher no. 20120623L2) was deposited with the State Key Laboratory of Phytochemistry and Plant Resources in West China, Kunming Institute of Botany, Chinese Academy of Sciences.

### General Experimental Procedures

Optical rotations were measured on a JASCO P-1020 digital polarimeter (JASCO, Tokyo, Japan). UV spectra were recorded using a Shimadzu UV-2401A spectrophotometer (Shimadzu, Kyoto, Japan). IR spectra were obtained on a Tensor 27 (Bruker Optics, Ettlingen, Germany) spectrometer with KBr pellets. 1D and 2D NMR spectra were performed on Bruker AM-400, DRX-500, or AVANCE III-600 spectrometers with TMS as an internal standard (Bruker Optics, Ettlingen, Germany). ESIMS were recorded on an an Agilent 6530 Q-Tof spectrometer (Agilent, Palo Alto, CA, USA). HREIMS were measured using a Waters Auto Premier P776 spectrometer (Waters, Milford, MA, USA). Column chromatography (CC) was performed using silica gel (100–200 and 200–300 mesh, Qingdao Marine Chemical Co. Ltd., Qingdao, China), MCI gel (CHP 20P, 75–150 μm; Mitsubishi Chemical Corporation, Tokyo, Japan), and Sephadex LH-20 (GE healthcare Bio-sciences AB, Sala, Sweden). Thin-layer chromatography (TLC) was carried out on silica gel 60 F254 on glass plates (Qingdao Marine Chemical Co. Ltd. Qingdao, China) using various solvent systems and spots were visualized by spraying improved Dragendorff’s reagent to the silica gel plates.

### Extraction and Isolation

The air-dried whole herb of *P. carinatus* (2 kg) was extracted with 70 % EtOH (24 h × 3), and the extract was partitioned between EtOAc and 10 % HCl/H_2_O. Water-soluble materials, after being adjusted at pH 10 with sat. Na_2_CO_3_, were then partitioned with CHCl_3_. CHCl_3_-soluble materials (17 g) were subjected to reversed-phase MPLC (RP-18) (MeOH/H_2_O, 10–95 %) to give fractions I–VIII.

Fr. I (2 g) was separated over a silica gel column (acetone/MeOH/diethylamine, 40:1:1–10:10:1) and purified by a Sephadex LH-20 column (MeOH) to afford lycodoline (30 mg). Fr. II (1.73 g) was subjected to repeated silica gel columns (acetone/MeOH/diethylamine, 40:1:1–10:10:1 and then petroleum ether/acetone/diethylamine, 50:40:1–10:80:1) to afford compound **1** (6 mg). Fr. III (4.78 g) was chromatographed over a silica gel column (CHCl_3_/MeOH/H_2_O, 90:10:1–50:50:1) to give three fractions (Fr. III–I to Fr. III–III). Fr. III–I was purified by sequential silica gel columns (petroleum ether/actone, 4:1) to get phlegmariurine A (3 mg). Fr. III–II was subjected to repeated silica gel columns eluted with petroleum ether/acetone/diethylamine (50:40:1–10:80:1) to afford fawcettimine (10 mg), phlegmariurine B (6 mg) and lycopodine (11 mg). Fr. III–III was chromatographed over repeated silica gel columns (petroleum ether/acetone/diethylamine, 50:40:1–10:80:1) to yield lobscurinol (4 mg) and lucidioline (6 mg). Fr. IV (4.7 g) was subjected to a silica gel column (CHCl_3_/MeOH/H_2_O, 90:10:1–50:50:1) to afford fractions I–IV. Fr. IV–III was purified by sequential silica gel columns (EtOAc/MeOH, 100:1) to get 8-deoxy-13-dehydroserratinine (15 mg). Fr. V (2.4 g) was chromatographed over repeated silica gel columns (CHCl_3_/MeOH/H_2_O, 90:10:1–50:50:1) to give five fractions (Fr. V–I to Fr. V–V). Fr. V–I was subjected to Sephadex LH-20 column (MeOH) and then further purified by repeated silica gel columns (EtOAc/MeOH, 100:1 and then petroleum ether/acetone, 4:1) to afford **2** (3 mg). Fr. IV–II was chromatographed over repeated silica gel columns (EtOAc/MeOH, 100:1 and then CHCl_3_/MeOH/H_2_O, 90:10:1–50:50:1) to give lycopodatine C (6 mg) and gnidioidine (4 mg). Fr. IV–III was subjected to repeated silica gel columns eluted with petroleum ether/acetone/diethylamine (50:40:1–10:80:1) to yield four fractions (Fr. IV–III–I to Fr. IV–III–IV). Then Fr. IV–III–I was purified by a silica gel column (CHCl_3_/MeOH/H_2_O, 90:10:1–50:50:1) to afford lycoposerramine-Q (32 mg) and lycodine (8 mg). Fr. IV–V was subjected to a silica gel column (CHCl_3_/MeOH/H_2_O, 90:10:1–50:50:1) to give *N*-oxide-lycoflexine (20 mg). Fr. VI (0.97 g) was chromatographed over repeated silica gel columns (petroleum ether/acetone/diethylamine, 50:40:1–10:80:1) to afford fractions I–IV. Fr. VI–II was purified by a silica gel column (petroleum ether/acetone, 4:1) to get obscurinine (40 mg). Fr. VI–IV was purified by Sephadex LH-20 (MeOH) and sequential silica gel columns (petroleum ether/EtOAc/diethylamine, 18:1:1) to afford malycorins B (5 mg). Fr. VII (1.1 g) was subjected to repeated silica gel columns eluted with petroleum ether/acetone/diethylamine (50:40:1–10:80:1) to afford nine fractions (Fr. VII–I to Fr. VII–IX). Fr. VII–I was purified by a silica gel column (EtOAc/MeOH/diethylamine, 18:1:1) to yield lycoflexine (10 mg).

### Acetylcholinesterase Inhibition

Acetylcholinesterase (AChE) inhibitory activity of the compounds isolated was assayed by the spectrophotometric method developed by Ellman et. al. [17] with slightly modification. *S*-Acetylthiocholine iodide, *S*-butyrylthiocholine iodide, 5,5′-dithio-bis-(2-nitrobenzoic) acid (DTNB, Ellman’s reagent), AChE derived from human erythrocytes were purchased from Sigma Chemical. Compounds were dissolved in DMSO. The reaction mixture (totally 200 μL) containing phosphate buffer (pH 8.0), test compound (50 μM), and acetyl cholinesterase (0.02 U/mL), was incubated for 20 min (30 °C). Then, the reaction was initiated by the addition of 40 μL of solution containing DTNB (0.625 mM) and acetylthiocholine iodide (0.625 mM) for AChE inhibitory activity assay, respectively. The hydrolysis of acetylthiocholine was monitored at 405 nm every 30 s for 1 h. Tacrine was used as positive control with final concentration of 0.333 μM. All the reactions were performed in triplicate. The percentage inhibition was calculated as follows: % inhibition = (E − S)/E × 100 (E is the activity of the enzyme without test compound and S is the activity of enzyme with test compound).

### Carinatine A (**1**)

Colorless oil; [*α*]_*D*_^26.6^−94.4 (*c* 1.2, MeOH); UV (MeOH) *λ*_max_ (log *ε*): 192 (3.54), 245 (3.86) nm; IR (KBr) *ν*_max_ 3424, 2925 and 1630 cm^−1^; ^1^H and ^13^C NMR see Table 1. ESIMS *m*/*z* 257 [M + H]^+^; HREIMS *m*/*z* 256.1579([M]^+^ calcd for C_16_H_20_N_2_O, 256.1576).

### Carinatine B (**2**)

White powder; [*α*]_*D*_^24.6^−56.9 (*c* 1.5, MeOH); UV (MeOH) *λ*_max_ (log *ε*): 201 (3.10) nm; IR (KBr) *ν*_max_ 3425, 2927 and 1630 cm^−1^; ^1^H and ^13^C NMR see Table 1. ESIMS *m*/*z* 278 [M + H]^+^; HREIMS *m*/*z* 277.1667([M]^+^ calcd for C_16_H_23_NO_3_, 277.1678).
